# Supplementary material for: Exploring YouTube content creators’ perspectives on generative AI in language learning: Insights through opinion mining and sentiment analysis
Source: PLoS One. 2024 Sep 26;19(9):e0308096. doi: 10.1371/journal.pone.0308096 (PMC11426429; doi:10.1371/journal.pone.0308096)
Supplement: S1 Appendix — (DOCX) [file pone.0308096.s002.docx]

**Appendix A: Code of Sentiment Analysis**

{

"cells": [

{

"cell_type": "code",

"outputs": [],

"source": [

"import googleapiclient.discovery\n",

"import pandas as pd\n",

"def takeList(npt=\"\"):\n",

"\n",

" api_service_name = \"youtube\"\n",

" api_version = \"v3\"\n",

" DEVELOPER_KEY = \"XXXXXXXXXXXXXXXXXXXXXXXXXXXXXX\"\n",

"\n",

" youtube = googleapiclient.discovery.build(api_service_name, api_version, developerKey = DEVELOPER_KEY)\n",

" videos=[]\n",

" \n",

" querystring='intitle:(\"generative AI\" | \"ChatGPT\") & intitle:(\"language learning\")'\n",

" order=\"viewCount\"\n",

" response= youtube.search().list(\n",

" part=\"snippet\",\n",

" q=querystring,\n",

" type=\"video\",\n",

" order=order,\n",

" pageToken=npt\n",

" ).execute()\n",

" print(response['pageInfo']['totalResults'])\n",

" i=0\n",

" npt=\"\"\n",

" while response:\n",

" i+=1\n",

" for item in response['items']:\n",

" videos.append([\n",

" item['id']['videoId'],\n",

" item['snippet']['publishedAt'],\n",

" item['snippet']['channelId'],\n",

" item['snippet']['title'],\n",

" item['snippet']['description'],\n",

" item['snippet']['channelTitle'],\n",

" item['snippet']['liveBroadcastContent'],\n",

" 'https://www.youtube.com/watch?v='+item['id']['videoId']\n",

" ])\n",

" if 'nextPageToken' in response and i<30:\n",

" print(i, response['nextPageToken'])\n",

" response = youtube.search().list(\n",

" part=\"snippet\",\n",

" q=querystring,\n",

" type=\"video\",\n",

" order=order,\n",

" pageToken=response['nextPageToken']\n",

" ).execute()\n",

" else:\n",

" if 'nextPageToken' in response:\n",

" print(i,response['nextPageToken'])\n",

" break\n",

" df = pd.DataFrame(videos, columns=['videoId','published_at', 'channelId', 'title', 'description','channelTitle','liveBroadcastContent','link'])\n",

" return df"

],

"metadata": {

"collapsed": false

},

"id": "659b2b3f9eab3b98"

},

{

"cell_type": "code",

"outputs": [],

"source": [

"def sentiment_analysis(transcription):\n",

" response = openai.ChatCompletion.create(\n",

" model=\"gpt-4-0613\",\n",

" temperature=0,\n",

" messages=[\n",

" {\n",

" \"role\": \"system\",\n",

" \"content\": 'Respond in the JSON format for the following Youtube transcript about ChatGPT in Language Learning Video: {\"sentiment\": sentiment_classification1, sentiment_classification2 (if needed) and sentiment_score: (-1 to 1)}, where the possible sentiment_classification categories are Optimistic, Distrustful, Mixed, Analytical, Ethical, Biased, Futuristic, Neutral'\n",

" },\n",

" {\n",

" \"role\": \"user\",\n",

" \"content\": transcription\n",

" }\n",

" ]\n",

" )\n",

" return response['choices'][0]['message']['content']"

],

"metadata": {

"collapsed": false

},

"id": "484be129dc88c9c8"

},

{

"cell_type": "code",

"outputs": [],

"source": [

"from sklearn.feature_extraction.text import CountVectorizer\n",

"from sklearn.decomposition import LatentDirichletAllocation\n",

"cv=CountVectorizer(max_df=0.9,min_df=2,stop_words=\"english\")\n",

"dtm=cv.fit_transform(df2[\"srt\"])\n",

"LDA=LatentDirichletAllocation(n_components=4,random_state=42)\n",

"LDA.fit(dtm)\n",

"for index,topic in enumerate(LDA.components_):\n",

" print(f\"TOP 20 WORDS FOR {index}\")\n",

" print([cv.get_feature_names_out()[index] for index in topic.argsort()[-20:]])\n",

" print(\"\\n\\n\")\n"

],

"metadata": {

"collapsed": false

},

"id": "e9f5a2907d9dd75f"

},

{

"cell_type": "code",

"outputs": [],

"source": [

"from sklearn.metrics import silhouette_score\n",

"import matplotlib.pyplot as plt\n",

"best_num_topics = 0\n",

"best_silhouette_score = -1\n",

"num_topics_range = range(2, 11) \n",

"silhouette_scores = []\n",

"for num_topics in num_topics_range:\n",

" LDA = LatentDirichletAllocation(n_components=num_topics, random_state=42)\n",

" LDA.fit(dtm)\n",

" topic_results = LDA.transform(dtm)\n",

" df2.loc[:, f\"topic_{num_topics}\"] = topic_results.argmax(axis=1).copy()\n",

"\n",

" # Calculate silhouette score for this number of topics\n",

" silhouette_avg = silhouette_score(dtm, df2[f\"topic_{num_topics}\"])\n",

" silhouette_scores.append(silhouette_avg)\n",

"\n",

" # Check if this is the best silhouette score\n",

" if silhouette_avg > best_silhouette_score:\n",

" best_silhouette_score = silhouette_avg\n",

" best_num_topics = num_topics\n",

"print(f'Best Number of Topics: {best_num_topics}, Best Silhouette Score: {best_silhouette_score}')\n",

"\n",

"# Plot the silhouette scores\n",

"plt.plot(num_topics_range, silhouette_scores, marker='o', linestyle='-')\n",

"plt.title(\"Silhouette Score vs. Number of Topics\")\n",

"plt.xlabel(\"Number of Topics\")\n",

"plt.ylabel(\"Silhouette Score\")\n",

"plt.grid(True)\n",

"plt.show()"

],

"metadata": {

"collapsed": false

},

"id": "dd31bdf9ab4a236"

},

{

"cell_type": "code",

"outputs": [],

"source": [

"import pyLDAvis\n",

"\n",

"import pyLDAvis.lda_model\n",

"pyLDAvis.enable_notebook()\n",

"\n",

"\n",

"dash = pyLDAvis.lda_model.prepare(LDA, dtm, cv, mds='tsne')\n",

"dash"

],

"metadata": {

"collapsed": false

},

"id": "e9e7be629e5eee1f"

}

],

"metadata": {

"kernelspec": {

"display_name": "Python 3",

"language": "python",

"name": "python3"

},

"language_info": {

"codemirror_mode": {

"name": "ipython",

"version": 2

},

"file_extension": ".py",

"mimetype": "text/x-python",

"name": "python",

"nbconvert_exporter": "python",

"pygments_lexer": "ipython2",

"version": "2.7.6"

}

},

"nbformat": 4,

"nbformat_minor": 5

}
