# Supplementary material for: Exploring YouTube content creators’ perspectives on generative AI in language learning: Insights through opinion mining and sentiment analysis
Source: PLoS One. 2024 Sep 26;19(9):e0308096. doi: 10.1371/journal.pone.0308096 (PMC11426429; doi:10.1371/journal.pone.0308096)
Supplement: Attachment — Submitted filename: ResponseToEditor.docx [file pone.0308096.s003.docx]

We express our gratitude to the editor and reviewers for their valuable feedback and constructive criticism of our manuscript submitted to PLOS ONE. We have carefully considered each comment and recommendation provided by the editor and reviewers, and have revised our manuscript accordingly. To provide a clear and comprehensive understanding of how we addressed and incorporated both editor’s and reviewers' suggestions into the revised version of our article, we prepared a detailed table outlining each comment and the corresponding revisions made in the manuscript. Two documents were prepared for the purpose of responding to the editor and the reviewers. The following table presents our response to the editor.

|  | **Annotation** | **Response to the Editor** |
| --- | --- | --- |
| **Editor** | | **Authors’ Response** |
|  | Thank you for submitting this insightful paper. While the manuscript, "Exploring YouTube content creators' perspectives on generative AI in language learning: Insights through opinion mining and sentiment analysis," provides a valuable analysis of YouTube videos on generative AI in language learning, it requires significant revisions before it can be considered for publication despite the reviewers has recommend for Minor Revisions. Reviewers may have missed major details and seems need more rigorous review. | Thank you for your feedback and for the opportunity to revise our manuscript. We appreciate the insights provided and have addressed the all revisions to ensure the paper meets the publication standards. |
|  | Firstly, the introduction, while comprehensive, needs a broader discussion on various generative AI models beyond ChatGPT and a clear justification for the exclusive focus on ChatGPT. The research questions, especially RQ1, lack clarity and require rephrasing to make the expectations more explicit. | Thank you for your valuable feedback on our manuscript. We have revised the introduction section to include a broader discussion of various generative AI models beyond ChatGPT, highlighting their diverse applications (page 1-3).  We did not solely focus on ChatGPT; as evident from our search query, we included the term "generative AI" as well. However, we highlighted ChatGPT separately due to its widespread adoption and rapid rise (page 11).  In our introduction section, we highlighted the dominance of ChatGPT based on our literature review (pages 4 &6). Although we did not intend to focus solely on ChatGPT, our review revealed that many studies on generative AI and language learning also emphasized ChatGPT, showing its widespread usage and frequent study in this area.  Additionally, we have revised our research questions according to the comments from you and the reviewers (pages 10-11). |
|  | The methods section, though detailed, leaves several critical points ambiguous. The role of video comments in the analysis is not clear, and there is confusion about whether these comments were used to reflect the perspectives of content creators or general users. | In our explanation for model prompt, we mistakenly wrote "comment" instead of "transcription" (as you can see in the Python code in the appendix, it is actually "transcription"). This caused a misunderstanding. We have corrected this on page 14.  Comments were only counted and are presented in Figure 7. We did not analyze the sentiments of the comments. Throughout the paper, we underscore that we only present data regarding comment counts and did not conduct any sentiment analysis of the comments. |
|  | Additionally, the classification and sentiment analysis processes need more transparency. There is no mention of accuracy checks for the classification models used, and the absence of code in the supplementary materials undermines the reproducibility of the study. | The classification and sentiment analysis model of this study is based on GTP-4-0613. Although there are some studies which try to check the performance and accuracy of GPT-4 (Payne et al, 2024, Gertz et al, 2024), the exact accuary level of GPT-4 is only mentioned as high in Capabilities declaration research page of official Open AI web site (<https://openai.com/index/gpt-4-research/>). We included this explanation on page 13. Since this study is not about the performance detection of GPT, we did not check the accuracy of our sentiment classification with human experts. That would be subject of another study.  Payne, D. L., Purohit, K., Borrero, W. M., Chung, K., Hao, M., Mpoy, M., ... & Hill, V. (2024). Performance of GPT-4 on the American College of Radiology In-training Examination: Evaluating Accuracy, Model Drift, and Fine-tuning. *Academic radiology*.  Gertz, R. J., Dratsch, T., Bunck, A. C., Lennartz, S., Iuga, A. I., Hellmich, M. G., ... & Kottlors, J. (2024). Potential of GPT-4 for Detecting Errors in Radiology Reports: Implications for Reporting Accuracy. *Radiology*, *311*(1), e232714. |
|  | The results section does not adequately relate the research questions to the outcomes. Figures 6 and 7, for example, are confusing due to the unclear units of analysis and the lack of detail about what is being measured. | We corrected the mistake regarding our explanation for model prompt. We clarified that we conduct sentiment analysis only for YouTube video transcripts. In the revised version of our manuscript, we clarified that confusion on page 14. |
|  | Moreover, the sentiment analysis lacks transparency regarding the models and libraries used, such as whether VADER or NLTK was employed. The manuscript should provide examples and include the code in the appendix to enhance transparency. | For the sentiment classification, we did not used VADER or other NLTK methods. Although those are very popular before GPT LLM, we preferred to set the ChatGPT 4 on our analysis because of its popularity and big data design feature. You can see the execution progress Appendix A in the manuscript. |
|  | The discussion section is currently disorganized and fails to effectively highlight the impact of the study. It needs to be restructured to address each research question separately and clearly. The discussion should also include recommendations and limitations, which are currently missing. | Based on the reviewer's and your feedback, we have reorganized the discussion section to address each research question separately and clearly. We have also included recommendations and limitations related to each research question within the discussion section (pages 28-36). |
|  | To improve the paper, the authors should:   1. Broaden the introduction to include various generative AI models and justify the focus on ChatGPT. 2. Clarify and rephrase RQ1 to make the expectations explicit. 3. Clearly explain the role of video comments and whether they reflect content creators' or general users' perspectives. 4. Provide transparency in the classification and sentiment analysis processes, including accuracy checks and the inclusion of code.  Relate the results directly to the research questions and clarify the units of analysis in the figures. 5. Organize the discussion to address each research question separately, include recommendations and limitations, and end with a strong conclusion. | We have carefully considered each suggestion and made the following revisions to improve our manuscript:   1. We broadened the introduction to include a discussion of various generative AI models beyond ChatGPT and provided a clear justification for focusing on ChatGPT due to its widespread adoption and rapid rise in popularity. This change ensures a more comprehensive background for our study. 2. Revised RQs are as follows:   **RQ1:** How do YouTube videos about generative AI in language learning specifically address different language skills such as speaking, listening, reading, and writing?  **RQ2:** What is the distribution of sentiments (Optimistic, Distrustful, Mixed, Analytical, Ethical, Biased, Futuristic, Neutral) expressed in YouTube videos about the use of generative AI for language learning?  **RQ3**: How are YouTube video contents clustered in terms of the wording by using text mining methodologies?  **RQ4:** What are the results of cross-sectional evaluations of the clustering, classification, and sentiment analyses of YouTube video contents about generative AI in language learning?   3. In the methods section, we provided a detailed explanation of the role of video comments. We clarified that comments were counted but not analyzed for sentiment, focusing solely on video transcripts. We addressed the misunderstanding caused by a mistake in the model prompt, where "comment" was incorrectly used instead of "transcription," and we have corrected this error. 4. We enhanced transparency in the classification and sentiment analysis processes. We explained that we used GPT-4 for sentiment classification due to its popularity and extensive data design, as mentioned in the Capabilities Declaration research page of the official OpenAI website. We also included code examples in the appendix to improve reproducibility. 5. We reorganized the discussion section to address each research question separately, providing clear insights and emphasizing the impact of the study. We included recommendations and limitations related to each research question. |
